# Computer assistance in modern craniomaxillofacial surgery

**DOI:** 10.1515/iss-2023-2002

**Published:** 2023-09-28

**Authors:** Nils-Claudius Gellrich, Michael Ehrenfeld

**Affiliations:** Department of Oral and Maxillofacial Surgery, Hannover Medical School, Hannover, Germany; Department of Oral and Maxillofacial Surgery, Ludwig-Maximilians-University of Munich, Munich, Germany

“The days of using CT, MRI or cone-beam data sets for diagnostics only are over! A 3D-voxel-based data set is the starting point of a common-trunk of 3D-imaging data use”.

Modern medicine is widely based on the use of digital patient data [1, 2]. Especially 3D-imaging data play a crucial role in decision making and treatment planning for most health care professionals [3]. By now, these three-dimensional data are mostly analyzed by radiologists and other physicians for diagnostic purpose to provide a detailed view on the patient’s anatomy and the status of this actual disease only. But far more information can be raised and processed to the benefit of the overall patient care. Regardless of anatomical region and medical specialty, a 3D data set is individually linked to the patient displayed. Computer-assistance effectively allows using these data for a pathway of quality control before, during and after surgery or combined treatment, i.e. for:

– visualization (look, objectify, analyze, decide) [4], [5], [6], [7], [8]
– segmentation [8]
– surgical planning including virtual blueprints [4, 6], [7], [8]
– intraoperative navigation [8, 9]
– medical modelling [8, 10, 11]
– 3D bio-model printing [8, 10]

The reader of this special issue shall be attracted by the variety of options computer-assistance allows. Craniomaxillofacial surgery spear-heads the use of 3D data sets to allow for the extra use of these data sets beyond the level of diagnostics only, due to the fact that 3D imaging is nearly always an immanent element in treatment of congenital and acquired pathologies and deformities [4, 5, 8, 12]. Furthermore, these digital technologies contribute to an evidence-based approach of treatment for the individual patient [4, 8, 13]. Other than in orthopedic surgery, robotics does not play a significant role in computer-assisted craniomaxillofacial surgery yet [14]. Nevertheless, one combined request for all surgical specialties is that 3D data have to be fully and freely usable for a surgeon-driven pathway (common trunk). Prerequisite is a robust DICOM viewer, allowing to interact with the 3D-dataset: look, objectify, analyze and decide [8].

Apart from the benefit of using these voxel-based information to the benefit of the individual patient, there are many more positive aspects to add:

– specialist training [11, 15]
– undergraduate and postgraduate medical education [11, 15]
– language independent interface improvement between doctors and surgeons to patient (image-based communication) [5, 16]
– quantification of surgically intended and post-operatively acquired results of skeletal changes [6, 8, 17]

Virtual treatment planning, surgical simulation, 3D modelling and the use of patient specific implants will lead to more individualized and adequate medicine and allows for an improved patient health care [4, 6, 8, 13].

The term “computer-assisted surgery” is broadly used to refer to virtual planning, patient-specific implants, guided surgery, real-time navigation and robotics [14]. The purpose of this ISS edition is also to clarify the evolution of computer-assisted surgery over the last 25 years within the field of craniomaxillofacial surgery and to show the pros and cons of implementing this kind of digital technology. Today’s computer technology differs completely to what was available 25 years ago so even on consumer level, hardware requirements are fully sufficient to be used for computer-assisted applications in surgery.

The evolution of computer-assisted surgery parallels the development in 3D imaging, which was spiral-CT based in the beginning and today includes cone-beam CT data sets as well as MRI data sets [8, 12] on a routine basis. The quality of routine 3D imaging technology is sufficient to serve for all above mentioned computer-assisted planning steps, bio-modelling or navigation purposes [8, 12].

To create objects as bio-models from 3D data sets, apart from digital blueprints vs. virtual surgical plans, is a further improvement due to development in medical biotechnologies. Today’s 3D printers even allow the use of autoclavable resins to print within the medical institution itself [18]. Bio-models can range from the whole skull to only parts of the skull [18].

Parallel to this modern development of 3D printing polymer technology, selective laser melting for manufacturing of metallic patient-specific-implants has proceeded as well as the use of PEEK (poly-ether-ether-ketone), ceramics and other biomaterials [8, 19].

To achieve the appropriately designed patient-specific implants, the digital interface between surgeon and bio-medical engineer needed to be developed. 

In this issue, international surgeons with a long experience in applying computer-assistance to their treatment strategies demonstrate the benefit of modern computer-assisted technologies to the reader. Virtual planning and computer-assistance are discussed in the fields of oncological surgery and the interface between surgery and radiotherapy, orbital and midfacial reconstruction, mandibular resection and reconstruction, skull base surgery and congenital deformity treatment in cases of hemifacial microsomia [4, 6, 8, 12, 13, 16, 20].
